# A genome scan for *Plasmodium falciparum* malaria identifies quantitative trait loci on chromosomes 5q31, 6p21.3, 17p12, and 19p13

**DOI:** 10.1186/1475-2875-13-198

**Published:** 2014-05-28

**Authors:** Audrey Brisebarre, Brice Kumulungui, Serge Sawadogo, Alexandre Atkinson, Séverine Garnier, Francis Fumoux, Pascal Rihet

**Affiliations:** 1 INSERM, UMR1090 TAGC, Marseille F-13288, France; 2 Aix-Marseille Université, Marseille F-13288, France; 3 Université des Sciences et Techniques de Masuku, Institut National Supérieur d’Agronomie et de Biotechnologies, Franceville, Gabon; 4 University of Ouagadougou, UFR des Sciences de la Santé, Ouagadougou, Burkina-Faso; 5 UMR-MD3, Marseille F-13385, France

**Keywords:** *Plasmodium falciparum*, Mild malaria, Parasitemia, Genome wide scan, Microsatellite markers, Linkage, Genetic association

## Abstract

**Background:**

Genome-wide studies have mapped several loci controlling *Plasmodium falciparum* mild malaria and parasitaemia, only two of them being significant at the genome level. The objective of the present study was to identify malaria resistance loci in individuals living in Burkina Faso.

**Methods:**

A genome scan that involved 314 individuals belonging to 63 families was performed. Markers located within chromosomes 6p21.3 and 17p12 were genotyped in 247 additional individuals belonging to 55 families. The linkage and the association of markers with parasitaemia and mild malaria were assessed by using the maximum-likelihood binomial method extended to quantitative trait linkage and the quantitative trait disequilibrium test, respectively.

**Results:**

Multipoint linkage analysis showed a significant linkage of mild malaria to chromosome 6p21.3 (LOD score 3.73, P = 1.7 10^−5^), a suggestive linkage of mild malaria to chromosome 19p13.12 (LOD score 2.50, P = 3.5 10^−4^), and a suggestive linkage of asymptomatic parasitaemia to chromosomes 6p21.3 (LOD score 2.36, P = 4.9 10^−4^) and 17p12 (LOD score 2.87, P = 1.4 10^−4^). Genome-wide family-based association analysis revealed a significant association between three chromosome 5q31 markers and asymptomatic parasitaemia, whereas there was no association with mild malaria. When taking into account 247 additional individuals, a significant linkage of asymptomatic parasitaemia to chromosome 17p12 (LOD score 3.6, P = 2 10^−5^) was detected.

**Conclusion:**

A new genome-wide significant malaria locus on chromosome 17p12 and a new suggestive locus on chromosome 19p13.12 are reported. Moreover, there was evidence that confirmed the influence of chromosomes 5q31 and 6p21.3 as loci controlling mild malaria or asymptomatic parasitaemia.

## Background

Malaria remains a major problem of public health in over one hundred countries worldwide. According to the World Health Organization (WHO), the number of cases in 2010 reached 219 millions (95% CI 154–289 millions), among which over 660 000 (95% CI 490 000–836 000) were fatal [1]. The disease ranges from asymptomatic parasitaemia or mild malaria, to severe anaemia, severe respiratory distress, or cerebral malaria. Fatal outcome occurs nearly exclusively in patients infected with *Plasmodium falciparum* who progress to severe malaria. Many studies provide evidence for human genetic factors controlling the outcome of infection by *P. falciparum*[2]. In particular, several candidate genes have been associated with resistance against severe malaria, while two GWA studies confirmed the *HBB* locus as a major locus in *P. falciparum* severe malaria [3,4]. Linkage analyses pointed out some significant linkage on chromosomes 6p21-p23 and 10p15, and several suggestive linkage with mild malaria or parasitaemia on chromosomes 1p36, 2p25, 4q13-q21, 5p15-p13, 5q31-q33, 6p25.1, 6q15-q16, 9q34, 12q21-q22, 13q13, 20p12 and 20q11 [5-9]. It should be stressed that linkage of mild malaria to chromosome 6p21-p23 and linkage of asymptomatic parasitaemia to 5q31-q33 have been reported at least twice in humans; these two loci correspond respectively to *Char3* and *Char8* that control *Plasmodium chabaudi* in mice [10,11].

The present study reports results from a genome-wide scan and additional further testing of promising regions. The maximum-likelihood binomial method extended to quantitative trait linkage analysis (MLB-QTL) and the quantitative trait disequilibrium test (QTDT) were applied to search for genetic linkage and association in the presence of linkage with mild malaria and asymptomatic parasitaemia.

## Methods

### Subjects and phenotype determination

The initial study population consisted of 314 individuals belonging to 63 families living in an urban district of Bobo Dioulasso in Burkina Faso; the mean age of sibs was 13.6 ± 6.3 years (range 1–34 years). The additional study subjects live in a rural area, Logoforousso, a village to the south-west of Bobo-Dioulasso (Burkina Faso). The study population comprised 247 subjects from 55 nuclear families; the mean age of the sibs was 10.1 ± 4.7 (3–29 years). The populations and the areas of parasite exposure have been extensively described [7,12]. Informed consent was obtained individually from all participants or their parents. The protocol was approved by the regional and national medical authorities of Burkina Faso. Parasitaemia data and DNA were available for the initial and additional study subjects. In addition, febrile episodes were extensively recorded by active case detection during 24 months for the initial population. For patients with fever, a thick blood film was prepared by the standard procedures. Diagnosis of mild malaria attack was based on *P. falciparum* parasitaemia, fever (axillary temperature more than 37.5°C) and clinical symptoms (headache, aching, vomiting or diarrhoea in the children); in that case no threshold of parasitaemia was used. In the absence of classical symptoms of malaria, and once others pathologies could not be eliminated, only children (age < 15 years) with more than 5,000 parasites per μl and older subjects with more than 2,000 parasites per μl were considered as having had a malaria attack. Each episode of illness was treated according to the recommendation of the CNRFP (Centre National de Recherche et Formation sur le Paludisme) of Burkina Faso. Parasitaemia was checked at the end of the treatment. Subjects who presented at least one mild malaria attack during the survey were considered in the analysis affected, while the others were considered unaffected. Determination of parasitaemia was described in previous studies [7,12]. Briefly, each family was visited 20 times during the 24 months of the study in the initial population, and 24 times during 24 months in the additional one, and parasitaemia was measured. In addition, parasitaemia was measured during febrile episodes. The mean number of parasitaemia measurements per subject was 15.2 ± 5.1. Fingerprint peripheral blood samples were taken from all family members present and thick and thin blood films were stained with Giemsa. The parasite determination and numeration were established blindly from two independent readings. Only *P. falciparum* asexual forms were retained to determine parasitaemia. Parasitaemia was defined as the number of parasitized erythrocytes observed per μl in thin blood films.

Mean of adjusted asymptomatic parasitaemia was a logarithmic transformation of the parasitaemia adjusted for seasonal transmission [7,12], after excluding parasitaemia during febrile episodes. To take into account the seasonality of the transmission, the influence of the date of the visits on ln(1 + parasitaemia) (LP) was evaluated by one way analysis of variance. The mean LP observed during each visit was calculated. The individual LP was then corrected for the visit effect by subtracting from each individual LP the mean LP of the corresponding visit. Phenotypes were calculated using the SPSS software (SPSS, Boulogne, France). Logistic regression and linear regression analyses confirmed the effect of age on mild malaria and mean of adjusted asymptomatic parasitaemia, respectively. Logistic regression analysis that took into account the effect of age also showed a positive correlation between mild malaria and asymptomatic parasitaemia (*P* = 0.013). The residual of the logistic regression model and the one of the linear regression model were calculated for mild malaria and parasitaemia, respectively; the residuals were further used in linkage or association analyses. All the sibs (in full) were included in linkage analyses.

### Genotyping

Genome scan was performed by using 400 microsatellite markers (Panel MD-10, Applied Biosystems), with an average distance of 10 cM. Multiplex PCR was performed under standard conditions, and the products were analysed by using a ABI377 systems (Applied Biosystems). Additional genotypes that were previously characterized in the initial study population were used for chromosomes 6p21.3 (TNFb, TNFd, D6S291) and 5q31-q33 (D5S642, D5S2117, D5S393, D5S399, D5S658, D5S436, D5S2090, D5S636, D5S2012, and D5S487) [5,7]. Additional markers were genotyped in the initial population comprising 314 individuals for chromosomes 5q31-q33 (D5S490, IRF1 microsatellite, IL4 microsatellite, IL9 microsatellite, D5S2115, D5S2017, D5S1972, ADRB2 microsatellite, D5S673, D5S410, and IL12B microsatellite) and 17p12 (D17S1881, D17S1844, D17S1812, D17S1791, D17S945, D17S1875, D17S969, D17S1856, D17S839, D17S953, D17S1824, D17S1800, D17S1850, D17S933, and D17S946). Additional markers were genotyped in the additional study population comprising 247 individuals for chromosomes 6p21.3 (TNFb, TNFd, D6S291) and 17p12 (D17S1881, D17S1844, D17S1812, D17S1791, D17S945, D17S1875, D17S969, D17S1856, D17S839, D17S953, D17S1824, D17S1800, D17S1850, D17S933, and D17S946). Genotyping procedures have been described for 6p21.3 and 5q31-q33 microsatellite markers [5,12]. The additional microsatellite markers located on chromosome 17p12 were genotyped under standard conditions. Mendelian inconsistencies were checked using the PedCheck program [13], whereas unlikely genotypes were detected using the MERLIN program [14]. Genotypes creating inconsistencies were corrected after re-examining raw data and re-genotyping if necessary.

### Statistical analyses

Nonparametric multipoint linkage analyses were performed with the MLBGH 3.0 program, which uses the general framework of Genehunter program [15,16]. The maximum likelihood binomial (MLB) method, which is based on the binomial distribution of parental marker alleles among affected offspring, overcomes the common problem of multiple sibs by considering the sibship as a whole. The MLB method in model free linkage analysis of quantitative traits was used to assess linkage of the residuals to genetic markers. The quantitative trait method introduces a latent binary variable (0;1) that captures the linkage information between the observed quantitative trait and the marker. The fully non-parametric approach with no assumption on the distribution of the residual for mild malaria was applied. The residual values were divided into 10 consecutive equal subintervals with equal probabilities. The probability to have a 0 value was fixed at 0.95, 0.85, 0.75, 0.65, 0.55, 0.45, 0.35, 0.25, 0.15 and 0.05 for phenotypes belonging to the consecutive deciles (1**–**10). The approach with the assumption on the normal distribution was used for parasitaemia. The MLB statistics was expressed in terms of a MLB-LOD and a one-sided standard normal deviate, denoted ZMLB. According to Kruglyak and Lander recommendations [17], linkage was considered suggestive and significant when the LOD score was higher than 2.2 and 3.6, respectively. The former is defined as the LOD score that would expected to occur one time by chance in a whole genome scan; the later is defined as the LOD score that would expected to occur 0.05 times by chance in a whole genome scan.

Combined association and linkage analyses of quantitative traits were carried out using the orthogonal model released in the QTDT 2.6.1 program [18]. Variance components are used to construct a test that utilizes information from all available offspring. The orthogonal model partitions association into between and within family components, and allowed use of markers and phenotypes as covariates in analyses. Evidence of association can be evaluated by likelihood-ratio test (null hypothesis likelihood L0 vs alternative hypothesis likelihood L1). Asymptotically, the quantity 2(LnL1-LnL0) is distributed as χ2 with df equal to the difference in number of parameters estimated. A false discovery rate of 10% was applied to correct for multiple testing [19].

## Results

### Genomewide linkage or association analysis of mild malaria and asymptomatic parasitaemia

**Figure 1:**
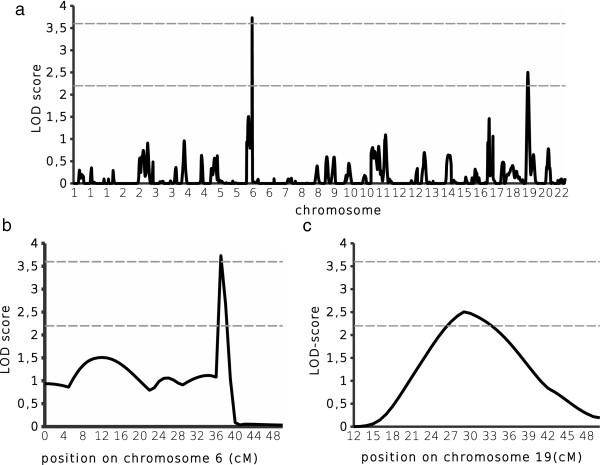
**Genomewide scan results of mild malaria.** A total of 314 individuals belonging to 63 nuclear families had been included in the analysis. Multipoint LOD scores implemented by MLBGH were plotted along the 22 autosomes **(a)**. The horizontal lines represent the threshold for a suggestive linkage (LOD score = 2.2) and a significant linkage (LOD score = 3.6). Detailed view of the regions with the highest LOD score on chromosomes 6 **(b)** and 19 **(c)**.

The genome scan was performed on 314 individuals belonging to 63 families. Figure 1 shows the multipoint LOD scores obtained for mild malaria. LOD scores indicating suggestive (LOD score > 2.2) or significant (LOD score > 3.6) linkage were achieved at 2 positions on chromosomes 6p21.3 and 19p13. A significant genome-wide LOD score and a suggestive genome-wide LOD score were obtained for mild malaria on chromosome 6p21.3 (LOD score = 3.73, P = 1.7 10^−5^) and on chromosome 19p13 (LOD score = 2.50, P = 3.5 10^−4^), respectively. The 1-LOD drop area for QTL location on chromosome 6p21.3 was very small, and included only 5 genes: *TNF, LTA, LTB, LST1,* and *NCR3*.

**Figure 2:**
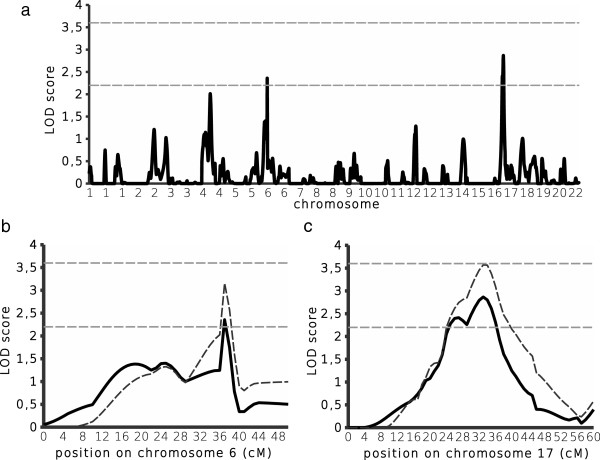
**Genomewide scan results of asymptomatic parasitaemia.** Multipoint LOD scores implemented by MLBGH were plotted along the 22 autosomes **(a)**. The horizontal lines represent the threshold for a suggestive linkage (LOD score = 2.2) and a significant linkage (LOD score = 3.6). Detailed view of the regions with the highest LOD score on chromosomes 6 **(b)** and 17 **(c)**. The primary genome scan results were based on 314 individuals belonging to 63 nuclear families (solid lines). The extended linkage results were based on 247 additional individuals belonging to 55 nuclear families, resulting in 561 individuals belonging to 118 families (dotted lines).

Figure 2 shows the LOD scores obtained for asymptomatic parasitaemia. Initial multipoint analyses revealed suggestive linkage on two chromosomes. For the 6p21.3 region that was significantly linked to mild malaria, we detected a suggestive linkage to asymptomatic parasitaemia (LOD score = 2.36, P = 4.9 10^−4^); the 1-LOD drop area for QTL location was very similar than the one for mild malaria, and contained the same genes*.* The best LOD score was achieved on chromosome 17p12 at D17S969/D17S799 (LOD score = 2.87, P = 1.4 10^−4^). The 1-LOD drop area for QTL location on chromosome 17p12 spanned 14 cM (D17S1875-D17S839), and contained 11 genes: *MAP2K4, ARHGAP44, MYOCD, ELAC2, COX10, DNAH9, HS3ST3B1, HS3ST3A1, ZNF18, CDRT15*, and *SHISA6*.

Combined linkage and association between malaria phenotypes and microsatellite markers were further tested. After applying a FDR of 10%, there was no significant association with mild malaria, whereas there was an association of asymptomatic parasitaemia with D5S642, the IL9 microsatellite, and D5S2017. Strikingly, those genetic markers are located within chromosome 5q31; noticeably, the distance between D5S642 and the IL9 microsatellite was 3.7 cM, and the one between the IL9 microsatellite and D5S2017 was 6.7 cM, suggesting the existence of several distinct malaria resistance loci in this region.

### Linkage of asymptomatic parasitaemia to chromosomes 17p12 and 6p21.3

Additional individuals (n = 247) that belonged to 55 families were also genotyped for the microsatellite markers in chromosomes 6p21.3 and 17p12, resulting in a total of 118 families with 561 individuals genotyped. The additional genotyping led to higher LOD scores for chromosomes 6p21.3 (LOD score = 3.16, P = 7 10^−5^) and 17p12 (LOD score = 3.6, P = 2 10^−5^), as shown in Figure 2. The whole 1-LOD drop area for QTL location on chromosome 6p21.3 contained 5 genes: *TNF, LTA, LTB, LST1,* and *NCR3.* The whole 1-LOD drop area for QTL location on chromosome 17p12 spanned 13 cM (D17S969-D17S839), and contained the 11 genes identified in the primary analysis.

## Discussion

Genome-wide linkage and association approaches were used to identify loci controlling parasitaemia or mild malaria. Four hundred thirty three microsatellite markers were genotyped for 314 individuals belonging to 63 families, and genome-wide analyses were performed to assess linkage and association between markers and malaria phenotypes that were monitored for two years. Additional markers located within chromosomes 6p21.3 and 17p12 and genotyped in 247 individuals belonging to 55 families were included in the analysis to further assess the linkage of asymptomatic parasitaemia to those chromosomal regions. The linkage and association results point out the influence of chromosomes 6p21.3, 19p13, 5q31, and 17p12 on mild malaria or asymptomatic parasitaemia.

The linkage results strongly support chromosome 6p21.3 as a locus controlling mild malaria, and suggest the influence of the same locus on parasitaemia. It should be stressed that the linkage of mild malaria to chromosome 6p21.3 was not a replicating study, because it mostly corresponds to the study published [5]. However, the suggestive linkage of parasitaemia to chromosome 6p21.3 was a new result, and strengthens the result previously obtained. These results are in line with results obtained in an independent human population [20] and mice [21]. Noticeably, the 1-LOD drop area contained only five genes, and among them, *TNF, LTA,* and *NCR3* have been associated with mild malaria or parasitaemia [22-24]. Besides, the results suggest a linkage of mild malaria to chromosome 19p13. The 1-LOD drop area for QTL location on chromosome 19p13 contained 233 genes. Interestingly, chromosome 19p13 has been also found to be associated with severe malaria [3], whereas it has not been reported as a mild malaria locus so far.

The influence of chromosome 5q31, which has been reported [6-8,12,25,26], is further supported by our linkage and association results at the genome wide significance level in the initial population. It should be stressed, however, that the linkage analysis did show neither a significant nor a suggestive linkage between asymptomatic parasitaemia and chromosome 5q31-q33; it provided evidence for two small peaks of linkage, yet with an LOD score lower than 2.2, whereas only one peak of linkage has been detected in previous linkage studies [7,8,12]. In particular, the previous linkage analysis that was performed in the same population by using another statistical method yielded a large peak of linkage, which may correspond at least to 2 loci showing a weak linkage signal in the present study [7]. In the same way, the genome-wide linkage and association analysis yielded a significant association of asymptomatic parasitaemia with three chromosome 5q31 microsatellite markers, which were several cM distal to each other, suggesting the existence of several malaria resistance loci on chromosome 5q31. This is consistent with the association study performed in the additional population [12], although the genetic markers associated with parasitaemia in the additional population and those associated with parasitaemia in the initial population were different. This is also consistent with linkage analyses in mice [11] and genetic studies in humans showing an association of *IL3, IL4, IL13, IRF1,* and *ARHGAP26* with parasitaemia, mild malaria or severe malaria [6,27-30].

Finally, a new locus controlling parasitaemia was identified based on a genome-wide significant LOD score after adding new families in the study. The peak of linkage located within chromosome 17p12 was sharp, and the 1-LOD drop area contained 11 genes both in the initial population and the extended one, the mouse orthologs of which are within *Char8* controlling parasitaemia in mice infected by *P. chabaudi*[11]. Among them, *HS3ST3A1* and *HS3ST3B1* were considered candidate genes on the basis of their function and the location of their mouse orthologs within *Char8*, and were found to be associated with asymptomatic parasitaemia in a population that corresponded mostly to the additional population in the present study [31]; interestingly, the linkage analysis that was performed in the initial population provided a suggestive linkage of parasitaemia to chromosome 17p12. *HS3ST3A1* and *HS3ST3B1* encode 3-OST-3A1 and 3-OST-3B1, respectively, which are sulphotransferases involved in heparan sulphate (HS) synthesis. They catalyse the 3–0 sulfation of carbohydrates, which is the last step of the synthesis, and which reflects the completion of HS synthesis. The variation in human genes controlling the sulphation of HS may alter the binding of sporozoites to hepatocytes and their development in those cells, as it has been shown in mice infected by *P. berghei*[32]. Taken together, all these results suggest that the genes involved in HS synthesis and located on chromosome 17p12 play a major role in the control of parasitaemia. Alternatively, other genes located in the same chromosomal region may also influence parasitaemia, and the additive effect of several genes may explain the strong linkage of parasitaemia to chromosome 17p12.

This is the first report of a malaria resistance locus on chromosome 17p12, whereas it confirms previous loci on chromosomes 6p21.3 and 5q31. Although replication studies are needed, our results support the recent hypothesis that genetic variation within genes involved in HS synthesis strongly influences parasitaemia. This may provide new insights for developing new therapeutic strategies.

## Competing interests

The authors declare that they have no competing interests.

## Authors’ contributions

AB carried out the linkage and association analyses. BK and SW genotyped the microsatellite markers, and constructed the genotype database. AA and SG participated in genotyping. FF participated in the design of the study, and revised the results and the manuscript. PR performed the design of the study, supervised the experiments and the statistical analyses, participated in the statistical analyses and the interpretation of data, and wrote the manuscript. All authors read and approved the final manuscript.
